# Co-application of mineral and organic fertilizers under deficit irrigation improves the fruit quality of the Wonderful pomegranate

**DOI:** 10.7717/peerj.11328

**Published:** 2021-05-18

**Authors:** Khalid F. Almutairi, Mahmoud Abdel-Sattar, Ahmed M. Mahdy, Mohamed A. El-Mahrouky

**Affiliations:** 1 Department of Plant Production, College of Food and Agriculture Sciences, King Saud University, Riyadh, Saudi Arabia, Riyadh, Saudi Arabia; 2 Pomology Department, Faculty of Agriculture, Alexandria University, Alexandria, Egypt; 3 Soil and Water Sciences Department, Faculty of Agriculture, Alexandria University, Alexandria, Egypt; 4 Soil Science Department, College of Food and Agriculture Sciences, King Saud University, Riyadh, Saudi Arabia

**Keywords:** Deficit irrigation, Fruit quality, Mineral fertilizer, Organic fertilizer, Pomegranate

## Abstract

**Background:**

The aim of this study was to determine the individual and interactive effects of various irrigation regimes and fertilizer treatments on the quality of the Wonderful pomegranate cultivar.

**Methods:**

Two field experiments were conducted over two consecutive growing seasons (2018 and 2019) to determine the individual and interactive effects of various organic and mineral fertilizer treatments on the fruit quality of the Wonderful pomegranate under various irrigation conditions. A split-plot experimental design was used, in which the main plots included three levels of irrigation (100%, 80%, and 60% of evapotranspiration) while the subplots included five fertilizer treatments with different co-application ratios of mineral and organic fertilizers.

**Results:**

All tested physicochemical properties of the fruit were significantly affected by the irrigation treatment, with irrigation at 80% of evapotranspiration representing the best strategy for reducing water use and improving fruit quality. Moreover, the co-application of mineral and organic fertilizers had a significant effect on fruit quality, with 75% mineral + 25% organic fertilizer improving all of the physical and chemical properties of the fruit in both experimental seasons. Irrigation and the co-application of mineral and organic fertilizers also had a significant interaction effect on the physicochemical attributes of fruit, which further increased fruit quality.

**Conclusions:**

The co-application of organic and mineral fertilizers produced better quality pomegranate fruit than mineral fertilizer alone under deficit irrigation conditions. This technique could therefore be applied to improve the fruiting of horticultural trees in arid growing regions.

## Introduction

Pomegranate (*Punica granatum* L.), a popular fruit in tropical and subtropical regions and an important crop for newly reclaimed soils in Egypt, has attracted attention for its health benefits (Lansky & Newman, 2007). The acceptability of pomegranate fruit to processors and consumers depends on a combination of several quality characteristics related to its mechanical and physicochemical properties, e.g.,the attractiveness, smoothness, and color of the skin; the amount of cracking; the presence of small seeds in the aril; the incidence of fruit sunburn (which causes dark-brown to black discoloration of the affected skin area); and the content of sugars, anthocyanin, and vitamin C (Al-Said, Opara & Al-Yahyai, 2009; Hmid et al., 2018).

Although pomegranate is fairly drought resistant, it requires regular watering to produce a high yield and heavy fruit (Holland, Hatib & Bar-Yaakov, 2009; Taha, 2018). Nevertheless, some studies have shown that reduced irrigation regimes in combination with organic/mineral fertilization during the fruit growth period can not only reduce water use but also positively affect fruit quality characteristics such as taste (by an increase of total soluble solids (TSS)) and color (Crisosto et al., 1994; Torrecillas et al., 2000; Mpelasoka, Behboudian & Mills, 2001; Prez-Pastor et al., 2007; Parvizi & Sepaskhah, 2015). In contrast, extreme watering may adversely affect fruit quality indicators by increasing vegetative growth, which promotes nutritional imbalance (Herrero & Guardia, 1992).

In general, water availability is the most critical and limiting factor for expanding cultivation and plant production in arid and semiarid regions. Therefore, worldwide research attention has been directed toward identifying efficient strategies for reducing water use during crop irrigation (Quda, 2016). Deficit irrigation is one such strategy for improving water productivity (i.e.,the crop yield per m^3^ of applied water). Under regulated deficit irrigation, irrigation is applied below the maximum crop evapotranspiration rate (ETc) during certain noncritical stages of the crop cycle, whereas complete irrigation is used during the dormant period of the growing season; under sustained deficit irrigation, watering is reduced to a fraction of the ETc uniformly throughout the growing season (Ruiz-Sanchez, Domingo & Castel, 2010). Compared with full irrigation, scarcity irrigation during the flowering and fruit set stages has previously been shown to increase the quantity of pomegranate fruit per tree without having adverse effects on the fresh weight or yield (Intrigliolo et al., 2013), whereas sustained deficit irrigation increases the number of fruit (Intrigliolo et al., 2013), fruit peel redness and firmness, and the soluble solids content (Pea, Arts-Hernndez & Aguayo, 2013). Pomegranate trees can activate physiological mechanisms to regulate their water status under scarcity conditions (Galindo et al., 2014); consequently, several studies have investigated pomegranate fruit properties under deficit irrigation (Mellisho et al., 2012; Rodrguez et al., 2012; Intrigliolo et al., 2013; Mena et al., 2013; Galindo et al., 2014; Parvizi, Sepaskhah & Ahmadi, 2014; Centofanti et al., 2017) both at harvest time and in storage (Laribi et al., 2013; Pea, Arts-Hernndez & Aguayo, 2013).

Organic cultivation, which is considered a benign and sustainable alternative production method, can also improve pomegranate fruit production. For example, soil organic matter plays a vital role in improving the physicochemical attributes of fruits (Marathe et al., 2009); it has been shown to affect nutrient fluxes (Marathe et al., 2012) in sweet orange (*Citrus sinensis* L.) and increase microbial biomass in pomegranate (Marathe et al., 2010; Marathe et al., 2011; Marathe et al., 2017; Mir, Sharma & Kumar, 2015).

Therefore, for pomegranate with its the long maturation time, a more rational approach to organic cultivation, including the exploitation of various locally available organic materials such as farmyard manure, poultry manure, vermin compost, and green manure, could be practically implemented to help rejuvenate depleted soil fertility and enrich the pool of nutrients available to the plants, which could benefit the medicinal properties of the fruit and other plant parts. In addition, the co-application of modest amounts of mineral fertilizers with organic manure can provide nutrient requirements (Baghdadi et al., 2018).

Few studies have assessed the effects of irrigation regimes on the nutritional aspects of pomegranate fruit, the physiological and production responses of pomegranate trees to water stress, or the effects of coapplying organic and mineral fertilizers under scarcity irrigation (Holland, Hatib & Bar-Yaakov, 2009; Abdel-Sattar et al., 2021). Previous field studies in southeastern Spain have examined the effects of different deficit irrigation applications on yield performance (Intrigliolo et al., 2013; Abdel-Sattar et al., 2021) and some fruit composition characteristics during fruit growth (Mellisho et al., 2012). Recently, it was also shown that deficit irrigation in combination with organic manure/mineral fertilizer or organic manure alone could improve the marketable fruit of Wonderful pomegranate trees, which may be attributable to the organic manure reducing the water stress of the growing plants (Abdel-Sattar et al., 2021).

Building on these previous studies, the objective of the present study was to examine the separate and interactive effects of various irrigation regimes and fertilizer treatments on the quality of the Wonderful cultivar of pomegranate.

## Materials & Methods

### Experimental site and plant materials

The present study was conducted during two successive growing seasons in 2018 and 2019 on 6-year-old Wonderful pomegranate trees located at North Coast, Matrouh Governorate, Egypt. The trees were spaced 4 2.5 m apart under a surface drip irrigation system with two lines/tree row running parallel. At the beginning of the experiment, soil samples were collected from depths of 030, 3060, and 6090cm and analyzed for various physicochemical properties (Table 1).

**Table 1:** Physical and chemical properties of the experimental soil.

Soil depth (cm)	Texture (%)			pH	EC (dSm^1^)	CaCO_3_ (%)	OM (%)	Soluble cations (meqL^1^)				Soluble anions (meqL^1^)		
	Sand	Clay	Silt					Na^+^	Ca^2+^	Mg^2+^	K^+^	HCO${}_{3}^{-}$	Cl	SO${}_{4}^{2-}$
030	43.10	13.13	43.77	8.1	1.04	29.75	0.32	4.2	2.8	2.4	1.0	2.2	5.4	2.8
3060	26.60	22.17	51.23	7.8	1.08	18.95	0.28	4.3	3.1	2.6	0.8	2.1	5.8	2.9
6090	19.40	20.39	58.21	7.8	1.13	12.14	0.17	4.5	3.6	2.5	0.7	2.0	6.0	3.3

In each season, 100% mineral fertilizer was added weekly at a rate of 240 units of N, 192 units of K_2_O, 60 units of P_2_O_5_, 50 units of MgO, and 71.65 units of CaO per hectare from March 1 through to mid-September with the irrigation water, whereas 100% organic manure containing 0.07 units of P_2_O_5_, 0.24 units of N, 0.13 units of CaO, 0.07 units of MgO, 0.27 units of K_2_O was added at a rate of 19.35 kg tree^1^ in November. Organic manure analysis was performed and recorded using the standard procedures provided by Kehres (2003); these analyses revealed that the organic fertilizer applied in the current study contained (on average across seasons) 22% organic C, 44% organic matter, 0.38% P_2_O_5_, 1.40% K_2_O, 1.24% N, 0.36% MgO, 0.68% CaO, 4,500-ppm Fe, 125-ppm Zn, 44-ppm Cu, and 450-ppm Mn, while it had a moisture content of 11.4 db and a C:N ratio of 11:1. All trees in the experimental site received the usual agricultural management practices applied in pomegranate orchards according to cultural practices applied by the Ministry of Agriculture, Egypt.

Climatic data were obtained from a weather station. The everyday reference evapotranspiration rate was computed according to the FAO 56 PenmanMonteith equation (Allen et al., 1998) and utilized when making irrigation decisions. ETc was then calculated by multiplying the everyday reference evapotranspiration rate by the crop coefficient, which increased from an initial value of 0.32 in March to a maximum value of 0.74 in July, August, and September, according to Intrigliolo et al. (2011). The amount of full irrigation water applied annually was 5,720 m^3^ ha^1^ in 2018 (equating to 100, 200, 480, 560, 840, 840, 840, 840, 560, 240, 120, and 100 L tree^1^ in each month from January to December, respectively) and 5,358 m^3^ ha^1^ in 2019 (equating to 97, 115, 458, 535, 798, 798, 790, 794, 532, 229, 115, and 97 L tree^1^ in each month from January to December, respectively). Irrigation was applied on 2, 4, 8, 8, 12, 12, 12, 12, 8, 4, 2, and 2 occasions in each month from January to December, respectively.

In each season, the treatments were arranged in a randomized complete block design using the split-plot technique (3 levels of irrigation 5 fertilizer treatments 4 replicates 4 trees per replicate) with 240 trees per season. The main plots included three irrigation treatments: full irrigation, i.e., 100% ETc (I_1_), as a control; 80% ETc (I_2_); and 60% ETc (I_3_). The subplots included five fertilizer treatments: 100% mineral fertilizer (T_1_), 100% organic manure (T_2_), 75% mineral fertilizer + 25% organic manure (T_3_), 50% mineral fertilizer + 50% organic manure (T_4_), and 25% mineral fertilizer + 75% organic manure (T_5_).

### Parameter measurements

To determine a range of fruit quality characteristics, fruit samples were manually collected from each tree once fruit had reached the ripening stage and become fully colored, which occurred after the first week of October in each season based on inspection the eye.

### Physical properties

The physical properties of five fruit samples per tree were assessed. The fruit weight (g), peel weight (g), aril weight (g), fruit diameter (cm), fruit length (cm), fruit diameter (cm), shape index (fruit length / fruit diameter ratio), and fruit volume were measured, and the fruit peel and aril percentages were calculated. In addition, the fruit peel color was assessed at one point in the equatorial area of each fruit using a Minolita Chroma Meter Model CR-2000 (Osaka, Japan) to measure the L*, a*, and b* values (where L* denotes brightness (0 to 100 representing black to white), a* denotes the red/green value, and b* denotes the blue/yellow value) (Itle & Kabelka, 2009).

### Chemical properties

The chemical properties of five additional fruit samples per tree were assessed. To determine the percentage of TSS, the fruit arils were pressed to obtain juice and a digital refractometer (Atago Co., Tokyo, Japan) was used to measure TSS in degrees Brix at 20 C. In addition, five mL of the juice was used to define the titratable acidity expressed as percentage of citric acid according to AOAC (2005), and the TSS/acidity ratio was determined by dividing the TSS percentage by the acidity percentage. The vitamin C content of the fruit was determined in mg ascorbic acid per 100 mL of juice according to AOAC (2005). The percentages of total and reducing sugars in the juice were also determined according to AOAC (2005), and the percentage of no-nreducing sugars was calculated from the difference between these values. Finally, the anthocyanin content was determined in mg 100 mL^1^ using a pH differential technique with two buffer schemes according to Giusti, Rodrguez-Saona & Wrolstad (1999).

### Statistical analysis

The means of the various treatments were compared using the least significant difference test at the 0.05 probability level according to Snedecor & Cochran (1990). All data were statistically analyzed using SAS version 9.13 (SAS Institute, Cary, NC, USA).

## Results

### Effects of irrigation regimes on the physical properties of fruit

The whole-plot effects of three irrigation regimes on the physical properties of Wonderful pomegranate fruit grown in the 2018 and 2019 seasons are presented in Table 2. In general, all of the measured physical properties of Wonderful pomegranate fruit were significantly affected by the irrigation treatments in both years (*P* <0.05), with the application of 80% ETc (I_2_) having a positive effect on all physical properties relative to the effects observed with 100% (I_1_) and 60% (I_3_) of the water requirements (Table 2). In contrast, the application of 60% ETc significantly reduced the values of all physical properties. For example, in 2018, pomegranate fruit weights were 322.8, 315.55, and 282.05 g under I_2_, I_1_, and I_3_, respectively, while values for fruit peel, aril, volume, length, diameter, and shape index were 36.02%, 63.99%, 342 cm^3^, 7.75 cm, 8.91 cm, and 0.87, respectively, under I_2_. Similarly, in 2019, pomegranate fruit weights were 323.8, 314.8, and 280.75 g under I_2_, I_1_, and I_3_, respectively, while values for fruit peel, aril, volume, length, diameter, and shape index were 35.89%, 64.11%, 341.25 cm^3^, 7.82 cm, 8.61 cm, and 0.91, respectively, under I_2_. Results from both seasons indicated that among the three irrigation regimes, the 80% ETc (I_2_) application resulted in significantly higher total fruit weight and improvements in all other physical properties relative to the respective measures observed with the 100% and 60% ETc applications.

**Table 2:** Whole-plot effects of three irrigation regimes on the physical properties of Wonderful pomegranate fruit in the 2018 and 2019 growing seasons.

Season	Irrigation regime	Fruit weight (g)	Fruit peel (%)	Fruit aril (%)	Fruit volume (cm^3^)	Fruit length (cm)	Fruit diameter (cm)	Shape index
2018	I_1_	315.55b	39.94b	60.06b	314.00b	7.55b	8.77b	0.863b
	I_2_	322.80a	36.02c	63.99a	342.00a	7.75a	8.91a	0.871a
	I_3_	282.05c	50.05a	49.95c	301.55c	7.42c	8.50c	0.873a
	LSD_0.05_	0.67	0.14	0.14	2.06	0.03	0.01	0.005
2019	I_1_	314.80b	40.22b	59.78b	313.05b	7.61b	8.73a	0.873b
	I_2_	323.80a	35.89c	64.11a	341.25a	7.82a	8.61b	0.908a
	I_3_	280.75c	50.40a	49.61c	296.95c	7.31c	8.36c	0.874b
	LSD_0.05_	0.66	0.11	0.11	2.51	0.03	0.07	0.006

**Notes.**

I_1_: 100% ETc; I_2_: 80% ETc; I_3_: 60% ETc. Mean values within a column for a particular season with different lowercase letters are significantly different at *P*0.05.

The main effects of the irrigation regimes on the skin color of Wonderful pomegranate fruit in the 2018 and 2019 seasons are listed in Table 3. In general, all skin color parameters were significantly influenced by the irrigation regimes in 2018 (*P* <0.05), with the application of 80% ETc (I_2_) having a positive effect on all parameters compared with the effects from the 100% (I_1_) and 60% (I_3_) water applications. In contrast, the application of 60% ETc significantly reduced all skin color parameters. The results from 2019 confirmed those from 2018, i.e., I_2_ also had a positive effect on skin color in 2019 (Table 3). As specific examples, the values of L* were 59.05, 54.83, and 48.53 in the first season and 58.72, 55.33, and 48.11 in the second season under I_2_, I_1_, and I_3_, respectively. The values of a* were 50.16, 45.66, and 42.66 in the first season and 49.97, 46.13, and 42.33 in the second season under I_2_, I_1_, and I_3_, respectively. The values of b* were 23.44, 23.81, and 23.89 in the first season and 23.32, 24.24, and 23.80 in the second season under I_2_, I_1_, and I_3_, respectively.

**Table 3:** Whole-plot effects of three irrigation regimes on the skin color of Wonderful pomegranate fruit in the 2018 and 2019 growing seasons.

Irrigation regime	2018			2019		
	L*	a*	b*	L*	a*	b*
I_1_	54.83b	45.66b	23.81a	55.33b	46.13b	24.24a
I_2_	59.05a	50.16a	23.44a	58.72a	49.97a	23.32b
I_3_	48.53c	42.66c	23.89a	48.11c	42.33c	23.80ab
LSD_0.05_	0.21	0.34	0.45	0.44	0.64	0.60

**Notes.**

I_1_: 100% ETc; I_2_: 80% ETc; I_3_: 60% ETc. Mean values within a column for a particular season with different lowercase letters are significantly different at *P*0.05.

### Effects of irrigation regimes on the chemical properties of fruit

**Figure 1:**
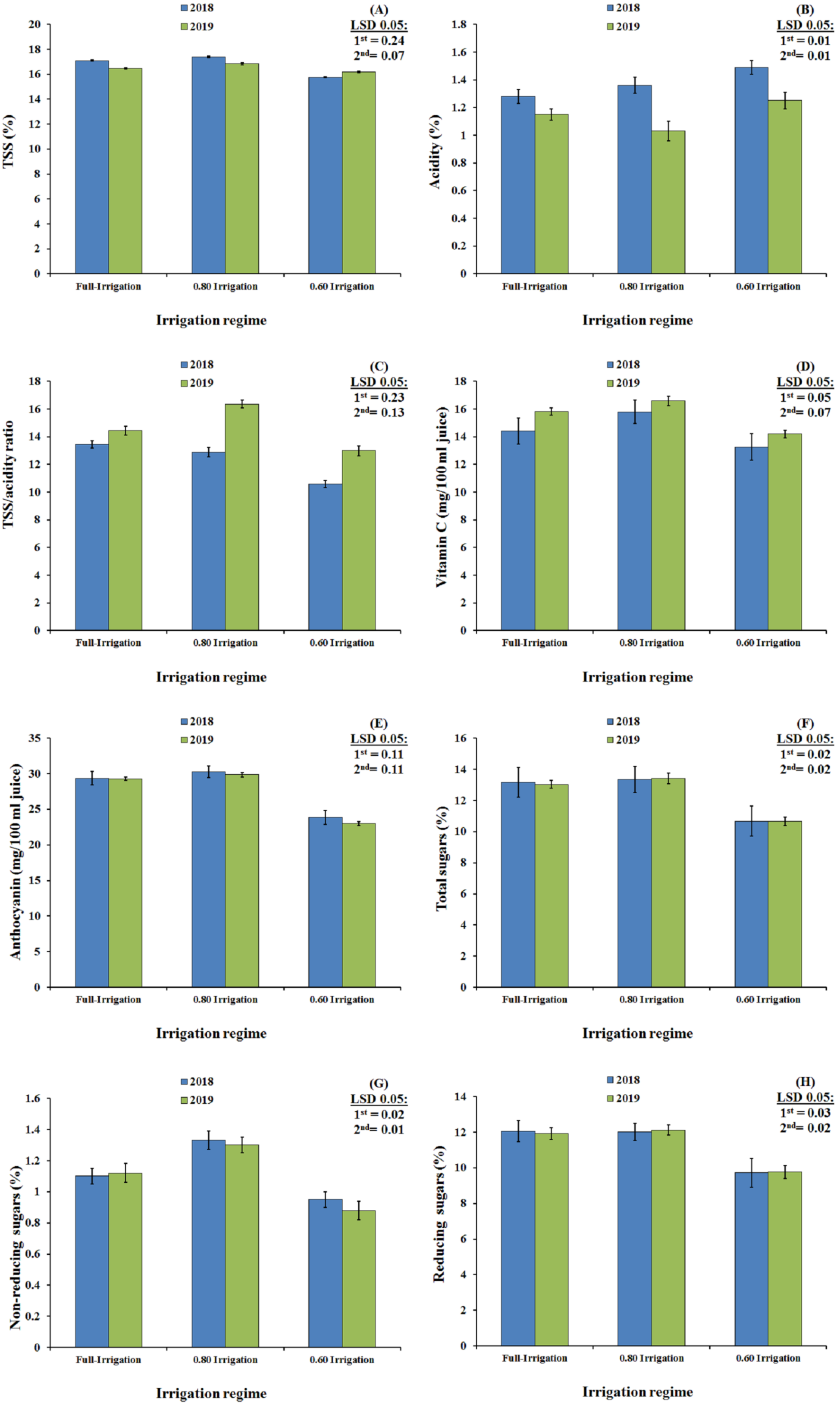
The interaction effect of irrigation and organic/mineral fertilizers on the fruit properties (A, TSS; B, Acidity; C, TSS/acidity ratio; D, Vitamin C; E, Anthocyanin; F, Total sugars; G, Non-reducing sugars; H, Reducing sugars) of Wonderful pomegranate in the 2018 and 2019 growing seasons.

The main effects of the irrigation treatments on the chemical properties of Wonderful pomegranate fruit in the 2018 and 2019 seasons are shown in Fig. 1. All of the chemical attributes were significantly affected by the irrigation treatments in 2018 (*P* <0.05), with the application of 80% ETc (I_2_) having a positive effect on all chemical properties except acidity, TSS/acidity, and reducing sugars when compared with the effects obtained with the 100% ETc (I_1_) and 60% ETc (I_3_) applications. Contrastingly, the application of 60% ETc significantly increased acidity and decreased all other chemical properties in the fruit. Similar results were obtained in 2019, i.e., 80% ETc (I_2)_ significantly increased the values of all chemical properties except for the acidity and reducing sugar content of the fruit (Fig. 1). Across the two seasons, the 80% ETc (I_2_) application had a higher positive effect on all chemical properties of pomegranate fruit except acidity, TSS/acidity, and reducing sugars than did the 100% ETc (I_1_) and 60% ETc (I_3_) applications.

### Effects of fertilizer treatments on the physical properties of fruit

The subplot effects of the fertilizer treatments on the physical properties of Wonderful pomegranate fruit grown in the 2018 and 2019 seasons are presented in Table 4. Generally, all of the physical properties were significantly affected by the fertilizer treatment in 2018 (*P* <0.05), with co-applications of mineral and organic fertilizers producing higher values for some physical property parameters compared with values obtained by applying 100% mineral fertilizer (T_1_). However, T_1_ produced higher values for fruit aril, volume, diameter, and length along with the same value for the shape index. The co-application of mineral and organic fertilizers also had a significant effect on all of the physical properties, with the combination of 75% mineral + 25% organic fertilizers (T_3_) giving the highest values for every property (Table 4). For example, the weights of the fruit were 311.83, 311.33, 305.33, 305.08, and 300.42 g under T_2_, T_3_, T_4_, T_5_, and T_1_, respectively, in 2018, whereas the values for fruit volume, peel, aril, length, diameter, and shape index showed similar patterns. Similar findings in 2019 confirmed the results of 2018 (Table 4). In both seasons, the T_2_ and T_3_fertilizer treatments produced higher positive effects on all physical properties than did the T_3_, T_4_, and T_5_ treatments.

**Table 4:** Sub-plot effects of five fertilizer treatments on the physical properties of Wonderful pomegranate fruit in the 2018 and 2019 growing seasons.

Season	Fertilizer treatment	Fruit weight (g)	Fruit peel (%)	Fruit aril (%)	Fruit volume (cm^3^)	Fruit length (cm)	Fruit diameter (cm)	Shape index
2018	T_1_	300.42c	40.61d	59.39b	317.75c	7.53c	8.72c	0.865b
	T_2_	311.83a	44.73a	55.27e	306.58d	7.46d	8.62d	0.865b
	T_3_	311.33a	40.42e	59.58a	331.58a	7.70a	8.82a	0.873a
	T_4_	305.33b	41.03c	58.97c	323.67b	7.64b	8.76b	0.873a
	T_5_	305.08b	43.22b	56.78d	317.83c	7.53c	8.71c	0.865b
	LSD_0.05_	0.86	0.17	0.17	1.30	0.04	0.02	0.006
2019	T_1_	298.67d	40.74d	59.26a	313.00c	7.49c	8.45b	0.886b
	T_2_	312.67a	44.76a	55.23d	301.33e	7.38d	8.34c	0.884b
	T_3_	310.75b	40.63d	59.38a	341.08a	7.82a	8.51b	0.919a
	T_4_	305.25c	41.33c	58.67b	320.67b	7.72b	8.74a	0.885b
	T_5_	304.92c	43.37b	56.63c	309.33e	7.48c	8.79a	0.852c
	LSD_0.05_	0.85	0.14	0.14	3.24	0.04	0.09	0.009

**Notes.**

T_1_: 100% mineral fertilizer; T_2_: 100% organic manure; T_3_: 75% mineral fertilizer + 25% organic manure; T_4_: 50% mineral fertilizer + 50% organic manure; T_5_: 25% mineral fertilizer + 75% organic manure. Mean values within a column for a particular season with different lowercase letters are significantly different at *P*0.05.

The subplot effects of the fertilizer treatments on the skin color parameters of Wonderful pomegranate fruit grown in the 2018 and 2019 seasons are presented in Table 5. In general, all of the skin color parameters were significantly affected by the fertilizer treatment in 2018 (*P* <0.05), with the application of 100% organic fertilizer (T_2_) significantly increasing all of the parameters except b* and a* (which decreased under T_2_) compared with the effects of applying 100% mineral fertilizer (T_1_). The co-application of mineral and organic fertilizers also had a significant effect on all skin color parameters, with the highest values being obtained when using the 75% mineral + 25% organic fertilizer treatment (T_3_) or 50% mineral + 50% organic fertilizer (T_4_) in that order (Table 5). Similar results from 2019 confirmed the 2018 data. For example, the values of b*, L* and a* under T_3_ were 22.73, 57.67, and 49.68, respectively, in 2018, and 22.61, 57.72, and 50.05, respectively, in 2019. Thus, for the effects on skin color parameters, T_3_>T_4_>T_2_c= T_5_>T_1_.

**Table 5:** Sub-plot effects of the five fertilizer treatments on the skin color of Wonderful pomegranate fruit in the 2018 and 2019 growing seasons.

Fertilizer treatment	2018			2019		
	L*	a*	b*	L*	a*	b*
T_1_	49.95d	44.57d	25.36a	49.91d	44.67c	25.46a
T_2_	53.52c	43.10e	22.32c	53.46c	42.95d	22.54c
T_3_	57.67a	49.68a	22.73c	57.72a	50.05a	22.61c
T_4_	56.12b	47.75b	23.36b	56.06b	47.92b	23.48b
T_5_	53.40c	45.69c	24.79a	53.11c	45.12c	24.87a

**Notes.**

T_1_: 100% mineral fertilizer; T_2_: 100% organic manure; T_3_: 75% mineral fertilizer + 25% organic manure; T_4_: 50% mineral fertilizer + 50% organic manure; T_5_: 25% mineral fertilizer + 75% organic manure. Mean values within a column for a particular season with different lowercase letters are significantly different at *P*0.05.

### Effects of fertilizer treatments on the chemical properties of fruit

The subplot effects of the fertilizer treatments on the chemical properties of Wonderful pomegranate fruit grown in the 2018 and 2019 seasons are presented in Table 6. In general, all of the chemical properties were significantly influenced by the fertilizer treatment in 2018 (*P* <0.05), with the application of 100% organic fertilizer (T_2_) or the co-application of organic and mineral fertilizers (T_3_ and T_4_) significantly increasing all chemical properties except acidity, which decreased significantly. In contrast, the application of 100% mineral fertilizer (T_1_) generated lower values for all of the chemical properties, except acidity, than the values obtained with treatments containing organic fertilizer (Table 6). The co-application of mineral and organic fertilizers had a significant effect on all of the chemical properties, with the highest values for all parameters, except acidity and TSS/acidity ratio, being obtained in both seasons with the 75% mineral + 25% organic fertilizer (T_3_), 50% mineral + 50% organic fertilizer (T_4_), and 100% organic fertilizer (T_2_) treatments in that order (Table 6); thus, co-application treatments improved the quality of pomegranate fruit. In general, the effects of the treatments on all chemical properties except acidity followed T_3_>T_4_>T_2_= T_5_>T_1_.

**Table 6:** Sub-plot effects of five fertilizer treatments on the chemical properties of Wonderful pomegranate fruit in the 2018 and 2019 growing seasons.

Season	Fertilizer treatment	TSS (%)	Acidity (%)	TSS/acidity ratio	Vitamin C (mg 100 mL^1^ juice)	Anthocyanin (mg 100 mL^1^)	Total sugars (%)	Non-reducing sugars (%)	Reducing sugars (%)
2018	T_1_	16.63c	1.47a	11.43d	14.32c	27.12d	12.24c	1.07c	11.17c
	T_2_	15.95d	1.24e	12.99a	13.83e	26.32e	12.04d	1.03d	11.02d
	T_3_	17.48a	1.43b	12.27c	15.18a	29.67a	12.82a	1.26a	11.56a
	T_4_	17.11b	1.41c	12.19c	14.80b	28.72b	12.64b	1.19b	11.46a
	T_5_	16.56c	1.33d	12.57b	14.25d	27.27c	12.23c	1.09c	11.14c
	LSD_0.05_	0.31	0.01	0.30	0.07	0.14	0.02	0.02	0.03
2019	T_1_	16.77b	1.24a	13.65e	15.38c	26.66d	12.16d	1.05c	11.11d
	T_2_	15.68e	1.02e	15.50a	14.57e	26.35e	12.02e	1.01d	11.01e
	T_3_	17.04a	1.21b	14.17d	16.52a	28.96a	12.80a	1.21a	11.59a
	T_4_	16.66c	1.14c	14.72c	15.94b	28.00b	12.62b	1.15b	11.47b
	T_5_	16.33d	1.11d	14.90b	15.20d	26.87c	12.21c	1.06c	11.14c
	LSD_0.05_	0.09	0.01	0.17	0.09	0.15	0.03	0.02	0.03

**Notes.**

T_1_: 100% mineral fertilizer; T_2_: 100% organic manure; T_3_: 75% mineral fertilizer + 25% organic manure; T_4_: 50% mineral fertilizer + 50% organic manure; T_5_: 25% mineral fertilizer + 75% organic manure. Mean values within a column for a particular season with different lowercase letters are significantly different at *P*0.05.

### Interaction effects of irrigation and fertilizer treatments in relation to the physical properties of fruit

**Figure 2:**
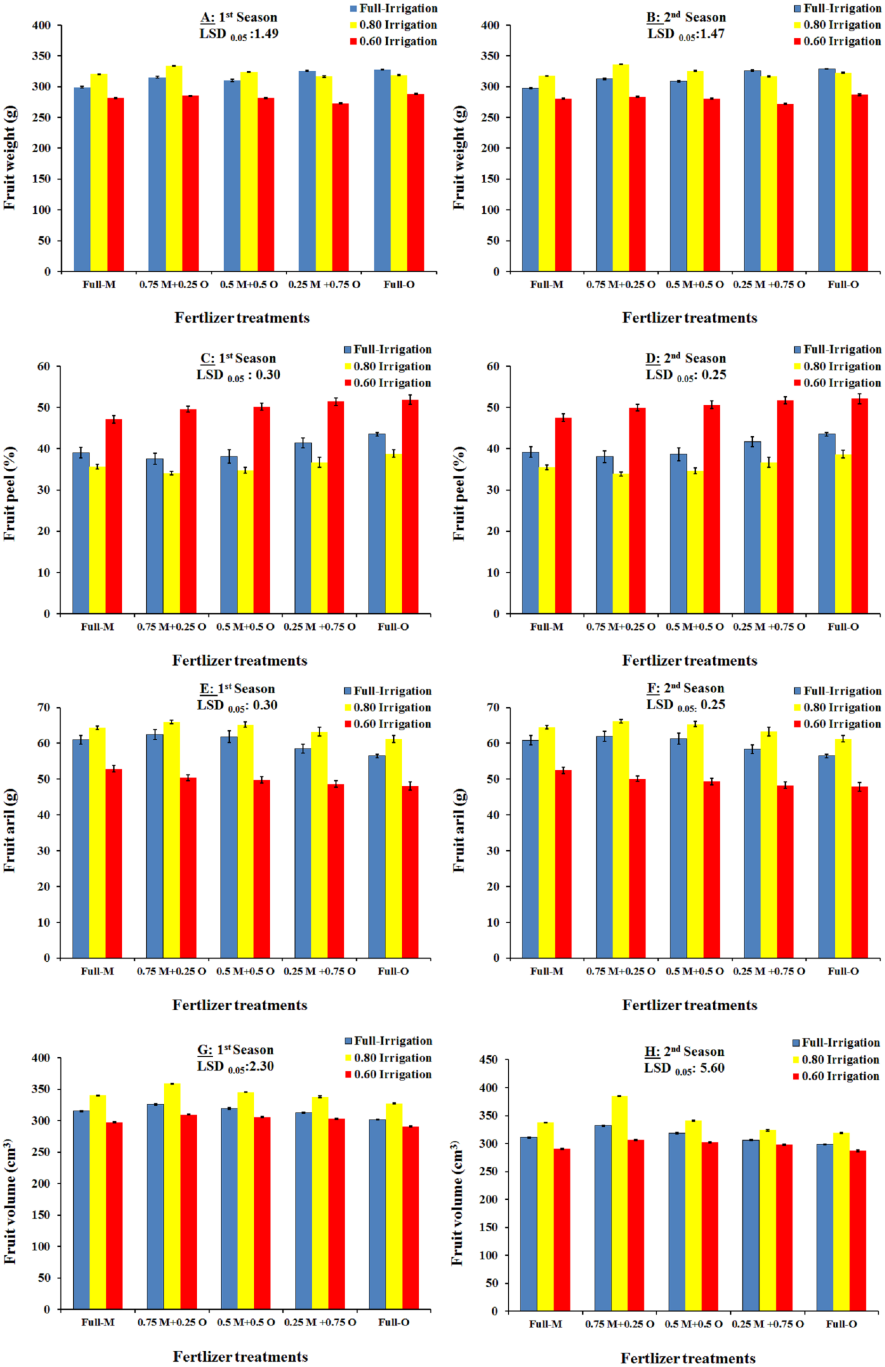
The interaction effect of irrigation and organic/mineral fertilizers on the fruit properties (Fruit weight: A and B; Fruit peel: C and D; Fruit aril: E and F; Fruit volume: G and H) of Wonderful pomegranate in the 2018 and 2019 growing seasons.

**Figure 3:**
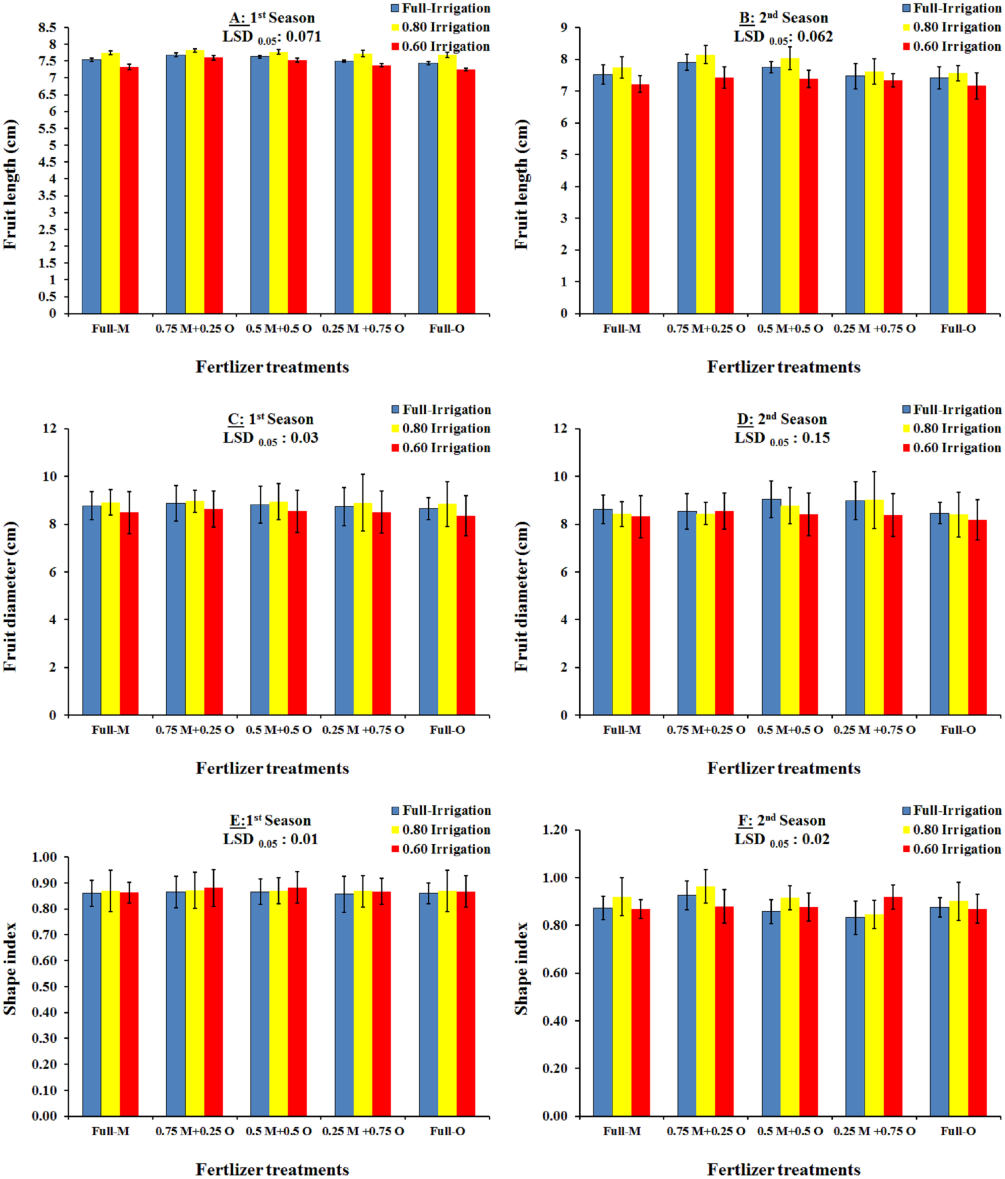
The interaction effect of irrigation and organic/mineral fertilizers on the geometric fruit properties (Fruit length: A and B; Fruit diameter: C and D; Shape index: E and F) of Wonderful pomegranate in the 2018 and 2019 growing seasons.

The two-way interactions between the irrigation regimes and fertilizer treatments on the physical properties of Wonderful pomegranate fruit grown in the 2018 and 2019 seasons are shown in Figs. 2 and 3. In 2018, the highest fruit weight was recorded with I_2_+T_3_ (333.75 g), followed by I_1_+T_2_ (327.75 g), and then I_1_+T_5_(325.50 g). In addition, the % fruit peel was significantly affected by the interaction between the irrigation regimes and fertilizer treatments, with the lowest percentages (and therefore the highest quality) being observed with the I_2_+T_3_ and I_2_+T_4_ treatments. These findings indicate that the use of organic fertilizer alone or in combination with mineral fertilizer enhanced the water content of the soil under deficit irrigation, which allowed the water supply to be reduced to 80% of the total requirement of the plants. The aril %, length, diameter, and volume of the fruit were also significantly affected by the interaction between the irrigation regimes and fertilizer treatments (Figs. 2 and 3), with higher values and therefore higher fruit quality being observed with the I_2_+T_3_ and I_2_+T_4_ treatments. The findings for 2018 were in agreement with the results from the 2019 season (Figs. 2 and 3).

The interaction effects of the irrigation regimes and fertilizer treatments on the skin color parameters of Wonderful pomegranate fruit grown in 2018 and 2019 are presented in Table 7. In 2018, the highest skin color (except b*) parameter values were observed with the I_2_+T_3_treatment, followed by the I_2_+T_4_ and I_1_+T_3_ treatments.

**Table 7:** Interaction effects of three irrigation regimes and five fertilizer treatments on the skin color parameters of Wonderful pomegranate fruit in the 2018 and 2019 growing seasons.

Treatment	2018			2019		
	L*	a*	b*	L*	a*	b*
I_1_+T_1_	52.47	46.02	25.22	52.76	46.58	25.44
I_1_+T_2_	54.77	43.53	23.57	55.65	43.50	23.78
I_1_+T_3_	56.71	48.00	22.82	57.33	48.80	23.31
I_1_+T_4_	56.03	46.09	23.64	56.49	46.42	24.25
I_1_+T_5_	54.18	44.68	23.78	54.42	45.36	24.47
I_2_+T_1_	52.17	48.94	24.75	52.07	49.70	24.83
I_2_+T_2_	60.03	46.13	22.61	59.54	45.78	23.05
I_2_+T_3_	63.46	54.70	22.23	63.52	54.69	21.76
I_2_+T_4_	62.06	51.32	23.03	61.78	51.38	22.77
I_2_+T_5_	57.53	49.70	24.59	56.67	48.30	24.22
I_3_+T_1_	45.23	38.76	26.12	44.89	37.75	26.12
I_3_+T_2_	45.76	39.65	20.76	45.18	39.58	20.80
I_3_+T_3_	52.86	46.36	23.14	52.32	46.66	22.75
I_3_+T_4_	50.29	45.85	23.41	49.92	45.98	23.41
I_3_+T_5_	48.51	42.70	25.99	48.24	41.71	25.91
LSD_0.05_	0.48	0.76	1.01	0.98	1.43	1.35

**Notes.**

I_1_: 100% ETc; I_2_: 80% ETc; I_3_: 60% ETc; T_1_: 100% mineral fertilizer; T_2_: 100% organic manure; T_3_: 75% mineral fertilizer + 25% organic manure; T_4_: 50% mineral fertilizer + 50% organic manure; T_5_: 25% mineral fertilizer + 75% organic manure.

### Interaction effects of irrigation regimes and fertilizer treatments in relation to the chemical properties of fruit

The interaction effects of the irrigation regimes and fertilizer treatments on the chemical properties of Wonderful pomegranate fruit grown in the 2018 and 2019 seasons are shown in Table 8. In 2018, the highest values for all chemical properties except acidity and the TSS/acidity ratio were observed with the I_2_+T_3_treatment, followed by I_2_+T_4_, and then I_2_+T_1_; these findings were confirmed by the results from 2019. These increases in chemical properties, such as increases in vitamin C, anthocyanin, TSS, reducing sugar, and no-nreducing sugar contents (but not acidity), were observed with all co-application treatments, following the order I_2_+T_3_>I_2_+T_4_>I_2_+T_1_ for all properties. However, although reducing the amount of water to 80% ETc improved the quality of the fruit, further reducing it to 60% ETc lowered fruit quality.

**Table 8:** Interaction effects of three irrigation regimes and five fertilizer treatments on the chemical properties of Wonderful pomegranate fruit in the 2018 and 2019 growing seasons.

Season	Treatment	TSS (%)	Acidity (%)	TSS/acidity ratio	Vitamin C (mg 100 mL^1^ juice)	Anthocyanin (mg 100 mL^1^)	Total sugars (%)	Non-reducing sugars (%)	Reducing sugars (%)
2018	I_1_+T_1_	17.23	1.36	12.71	14.21	29.45	13.16	1.11	12.05
	I_1_+T_2_	16.03	1.15	13.97	13.93	27.98	12.97	1.00	11.97
	I_1_+T_3_	17.70	1.32	13.46	15.21	30.73	13.38	1.20	12.18
	I_1_+T_4_	17.43	1.31	13.30	14.63	29.80	13.26	1.15	12.11
	I_1_+T_5_	17.13	1.25	13.70	14.00	28.78	13.05	1.04	12.01
	I_2_+T_1_	17.40	1.45	12.05	15.85	30.08	13.35	1.34	12.01
	I_2_+T_2_	16.83	1.21	13.91	14.78	28.43	13.07	1.23	11.84
	I_2_+T_3_	18.10	1.43	12.66	16.60	32.48	13.69	1.42	12.27
	I_2_+T_4_	17.75	1.41	12.64	16.20	31.10	13.50	1.39	12.12
	I_2_+T_5_	16.90	1.29	13.1	15.51	29.15	13.15	1.28	11.87
	I_3_+T_1_	15.28	1.61	9.52	12.91	21.83	10.22	0.77	9.45
	I_3_+T_2_	15.00	1.35	11.11	12.80	22.55	10.09	0.86	9.24
	I_3_+T_3_	16.65	1.56	10.70	13.73	25.80	11.38	1.15	10.23
	I_3_+T_4_	16.15	1.52	10.63	13.58	25.60	11.18	1.04	10.14
	I_3_+T_5_	15.65	1.44	10.91	13.23	23.88	10.48	0.94	9.54
	LSD_0.05_	0.53	0.024	0.51	0.12	0.23	0.042	0.038	0.056
2019	I_1_+T_1_	17.23	1.25	13.78	15.81	29.25	12.97	1.12	11.85
	I_1_+T_2_	15.63	0.98	16.03	14.78	28.30	12.85	1.02	11.83
	I_1_+T_3_	16.83	1.22	13.85	16.60	30.73	13.28	1.21	12.07
	I_1_+T_4_	16.59	1.17	14.23	16.20	29.63	13.18	1.16	12.02
	I_1_+T_5_	16.10	1.13	14.26	15.64	28.55	12.88	1.07	11.81
	I_2_+T_1_	17.30	1.14	15.21	16.70	29.83	13.37	1.30	12.07
	I_2_+T_2_	15.95	0.94	16.92	15.70	28.45	13.12	1.20	11.92
	I_2_+T_3_	17.50	1.12	15.63	17.28	31.48	13.79	1.41	12.39
	I_2_+T_4_	16.85	1.00	16.89	17.10	30.75	13.57	1.36	12.21
	I_2_+T_5_	16.58	0.97	17.09	16.13	28.75	13.23	1.24	11.99
	I_3_+T_1_	15.78	1.32	11.95	13.64	20.90	10.15	0.75	9.41
	I_3_+T_2_	15.45	1.14	13.56	13.25	22.30	10.10	0.82	9.28
	I_3_+T_3_	16.80	1.29	13.03	15.70	24.68	11.33	1.02	10.30
	I_3_+T_4_	16.55	1.27	13.03	14.53	23.63	11.13	0.95	10.18
	I_3_+T_5_	16.30	1.22	13.36	13.85	23.30	10.52	0.88	9.64
	LSD_0.05_	0.16	0.023	0.29	0.15	0.26	0.044	0.028	0.052

**Notes.**

I_1_: 100% ETc; I_2_: 80% ETc; I_3_: 60% ETc; T_1_: 100% mineral fertilizer; T_2_: 100% organic manure; T_3_: 75% mineral fertilizer + 25% organic manure; T_4_: 50% mineral fertilizer + 50% organic manure; T_5_ :25% mineral fertilizer + 75% organic manure.

## Discussion

Our results showed that 100% and 80% ETc applications significantly increased all physical properties of Wonderful pomegranate in the two tested seasons, whereas 60% ETc significantly reduced all physical properties. Thus, the water deficit is a limiting factor for Wonderful pomegranate fruit quality. Abdel-Sattar & Mohamed (2017) reported similar results, i.e., that deficit irrigation management was generally the main reason for reduced fruit volume and weight as well as shape index in Manfalouty pomegranate fruit. In addition, our results were in agreement with the findings of Rodrguez et al. (2012), Mellisho et al. (2012), Intrigliolo et al. (2013), Mena et al. (2013), Galindo et al. (2014), Parvizi, Sepaskhah & Ahmadi (2014), and Centofanti et al. (2017).

In the present study, all pomegranate skin color parameter values were significantly reduced with the 60% ETc application, indicating that a water deficit also affected the skin color of the fruit. Furthermore, deficit irrigation reduced the values of a* and b*, which is in agreement with the findings of Laribi et al. (2013), who observed redder and darker fruit at harvest under deficit irrigation. These skin color characteristics predominantly arise from an increase in anthocyanins and sugars and from reduced vegetative growth (i.e., nutritional status) (Intrigliolo et al., 2013; Laribi et al., 2013; Martnez-Nicols et al., 2019). Our results are also in agreement with those of Gelly et al. (2003) for peach, *Prunus persica* (L.) Batsch, and Martnez-Nicols et al. (2019) for the Wonderful and Mollar de Elche pomegranate cultivars.

Similarly, all of the skin color parameters in Wonderful pomegranate fruit grown in the 2018 and 2019 seasons were significantly affected by fertilizer treatment. Application of 100% organic fertilizer significantly increased all of the parameters except a* and b*, which were decreased, relative to the application of 100% mineral fertilizer. The co-application of mineral and organic fertilizers also had a significant effect on all skin color parameters, with the highest values being obtained with the 75% mineral + 25% organic fertilizer treatment. This may have been due to the organic fertilizer improving the physiochemical properties of the soil, e.g., aeration, water movement, water-holding capacity, cation exchange capacity, and availability of nutrients (Mahdy, 2012), which in turn resulted in improved skin color parameters. These findings agree with those reported in several other studies (e.g., Crisosto et al., 1994; Mpelasoka, Behboudian & Mills, 2001; Intrigliolo et al., 2013; Laribi et al., 2013; Martnez-Nicols et al., 2019).

The application of 100% and 80% ETc significantly decreased the chemical properties of the Wonderful pomegranate, whereas 60% ETc significantly increased acidity and decreased all other chemical properties. In 2019, the 80% ETc treatment significantly increased all chemical properties of fruit except acidity and reducing sugar content. In general, these results agree with those of Abdel-Sattar & Mohamed (2017), who reported that fruit quality indexes such as TSS/acidity ratio, TSS, and acidity, as well as anthocyanin and vitamin C content, increased under deficit irrigation in Manfalouty pomegranate fruit, whereas the acidity of the fruit juice decreased. Our results also support those reported by Laribi et al. (2013), Intrigliolo et al. (2013), and Martnez-Nicols et al. (2019) for other pomegranate cultivars.

When assessing the subplot effects of fertilizer treatments, we found that all of the physical properties were significantly affected by the fertilizer treatment in both seasons, with co-applications of mineral and organic fertilizers producing higher values for some parameters than were obtained with 100% mineral fertilizer alone. However, 100% mineral fertilizer produced higher fruit aril, volume, diameter, and length values with identical shape index values. Among the co-applications of mineral and organic fertilizers, a combination of 75% mineral + 25% organic fertilizers provided the highest value for every property. The improved physical and chemical characteristics achieved through co-application of mineral and organic fertilizers may be associated with the beneficial changes in soil P, K, and N, and in soil organic matter and organic matter-dependent soil properties, as well as the availability of nutrients (Mahdy, 2012; Baghdadi et al., 2018). Similarly, Marathe et al. (2017) reported that the application of organic fertilizers, such as farmyard manure and poultry manure, significantly enhanced pomegranate fruit yield and quality compared with the application of inorganic fertilizers due to the excess in nutrient availability. These results are also in line with those reported by Mellisho et al. (2012), Rodrguez et al. (2012), Intrigliolo et al. (2013), Mena et al. (2013), Galindo et al. (2014), Parvizi, Sepaskhah & Ahmadi (2014), and Centofanti et al. (2017), who associated the high-quality attributes of fruit with application of organic fertilizers.

Our results also showed that all of the measured chemical properties of Wonderful pomegranate fruit grown in 2018 and 2019 were significantly influenced by fertilizer treatment. Specifically, 100% organic fertilizer or the co-application of organic and mineral fertilizers significantly increased all chemical properties except acidity, which significantly decreased, whereas 100% mineral fertilizer generated lower values for all chemical properties except acidity. Among the co-application treatments, the highest values for all parameters except acidity and TSS/acidity ratio were obtained with the 75% mineral + 25% organic fertilizer treatment. The organic fertilizer therefore seems to have directly and indirectly enhanced the physiochemical properties of the soil and the availability of nutrients (Mahdy, 2012; Baghdadi et al., 2018; Abdel-Sattar et al., 2021). Similarly, Ray et al. (2014) reported that the fruit of pomegranate plants treated with 300-g N + 1-kg neem cake per plant showed high TSS (12.29Bx), total sugar (10.74%), reducing sugar (9.78%), non-reducing sugar (1.09%), and ascorbic acid (21.93 mg 100 mL^1^ juice) contents as well as low acidity (0.39%). Amin, Ali & El-Moneim (2017) also showed that a mixture of organic and biofertilizers (organic N and PK raw mineral rocky materials at 2,000 g plus NPK biofertilizer at 300 mL plant^1^) provided the greatest improvement in all of the studied growth parameters for pomegranate, with the promotion of mineral composition and total chlorophyll noted in young trees treated with mixed organic materials (NPK at 2,000 g plant^1^) and biofertilizers (NPK at 300 mL plant^1^). Furthermore, Aly & Zagzog (2019) found that the application of biofertilizer and organic fertilizer enhanced all vegetative growth parameters of Zaghloul date palm (*Phoenix dactylifera* L.), with the replacement of mineral fertilizer in this scenario also reducing pollution. Moreover, OlyaieTorshiz et al. (2017) suggested that the use of biofertilizer in combination with organic fertilizers, particularly granular humic acid, could be used in pomegranate orchard management to improve pomegranate yield and prevent crop losses resulting from cracking, nutrient deficiency, and *Ectomyelois ceratoniae* Zeller infestation. The findings of the present study were also in agreement with those reported by Intrigliolo et al. (2013), Mena et al. (2013), Galindo et al. (2014), Parvizi, Sepaskhah & Ahmadi (2014), and Centofanti et al. (2017) in relation to the enhancement of soil porosity and infiltration rate as well as water retention after organic fertilizer application.

Our findings indicate that the use of organic fertilizer alone or in combination with mineral fertilizer enhances the water content of the soil under deficit irrigation, allowing the amount of water supplied to be reduced to 80% of the total requirement of the plants. Specifically, fruit weight, aril %, length, diameter, and volume were significantly affected by the interaction between the irrigation regimes and fertilizer treatments. These findings were apparent in both growing seasons and were likely due to the organic fertilizer having a beneficial effect on water content and releasing more nutrients into the soil in an available form. Similar trends were observed by Intrigliolo et al. (2013), Parvizi, Sepaskhah & Ahmadi (2014), and Centofanti et al. (2017), who found that deficit irrigation is usually the principal reason for reduced fruit volume, length, diameter, weight, and aril.

The interaction effects of the irrigation regimes and fertilizer treatments also increased the skin color parameters (except b*) in Wonderful pomegranate fruit in both seasons, again because the application of mineral fertilizer in combination with organic fertilizer improved the water content of the soil under deficit irrigation, which released more nutrients into the soil in an available form, allowing for a reduction in amount of water supplied to pomegranate (80% of the total requirement). Similarly, Mena et al. (2013) found that reduced irrigation caused a dramatic reduction in bioactive phenolic compounds, particularly anthocyanin, in pomegranate fruit, which resulted in color changes to the juice (it became more yellowish). The results of Gelly et al. (2003) for peach, Laribi et al. (2013) for Mollar de Elche pomegranate, Abdel-Sattar & Mohamed (2017) for Manfalouty pomegranate, and Martnez-Nicols et al. (2019) for the Wonderful and Mollar de Elche pomegranate cultivars are also in agreement with our findings.

In both seasons, the chemical properties of Wonderful pomegranate fruit (with the exception of acidity and the TSS/acidity ratio) were improved via the interacting effects of the irrigation regimes and fertilizer treatments. This is likely because the organic fertilizers released more nutrients into the soil in an available form and had a beneficial effect on the water-holding capacity of the soil, again allowing the water supply to be reduced to 80% of the total requirement. The increase in chemical properties such as the vitamin C, anthocyanin, TSS, reducing sugar, and non-reducing sugar contents was observed with all co-application treatments. However, reducing the amount of water to 60% ETc resulted in lower quality fruit. These findings agree with those of Khattab et al. (2011) and Abdel-Sattar & Mohamed (2017), who concluded that deficit irrigation could improve vitamin C, anthocyanin, and TSS content, increase TSS/acidity ratio, and reduce acidity in pomegranate fruit. Similarly, Mena et al. (2013) found that reducing the irrigation levels to 43% and 12% of ET_0_ dramatically reduced the amount of bioactive phenolic compounds, particularly anthocyanin, in pomegranate fruit, which accordingly caused the color of the aril juice to become more yellowish.

Organic fertilizer has a micronutrient content that is several fold higher than that of mineral fertilizer, which leads to the mineralization of organically bound forms, the establishment of organic chelates of higher stability, or the production of stable water-soluble complexes, which have a reduced susceptibility to absorption, precipitation, and/or fixation (Mir, Sharma & Kumar, 2015; Shambhavi et al., 2020). Therefore, as previously mentioned the co-application of organic and mineral fertilizers increases the water-holding capacity of the soil under deficit irrigation and allows the water supply to the plants to be reduced (to 80% of the total requirement in the current study) (Kkyumuk, Yildiz & MerI, 2020).

## Conclusions

We examined the separate and interactive effects of various irrigation regimes and fertilizer treatments on the quality of the Wonderful cultivar of pomegranate. Our results suggest that, under deficit irrigation, the co-application of organic and mineral fertilizers produces better quality pomegranate fruit than does the application of mineral fertilizer alone. Reducing the water supply to 80% ETc improves fruit quality when compared with the fruit produced by 100% and 60% ETc applications. The best combination of mineral and organic fertilizers was 75% mineral + 25% organic fertilizers, which produced the highest values of fruit quality. Using this technique could promote the partitioning of metabolites, which would favor the fruiting of horticultural trees in arid growing regions.

##  Supplemental Information

10.7717/peerj.11328/supp-1 Data S1 Raw data

## Additional Information and Declarations

### Data Availability

The following information was supplied regarding data availability: The raw measurements are available in the Supplemental Files.
